# Transcriptome and gene expression analysis of *Rhynchophorus ferrugineus* (Coleoptera: Curculionidae) during developmental stages

**DOI:** 10.7717/peerj.10223

**Published:** 2020-11-02

**Authors:** Hongjun Yang, Danping Xu, Zhihang Zhuo, Jiameng Hu, Baoqian Lu

**Affiliations:** 1 College of Life Science, China West Normal University, Nanchong, Sichuan, China; 2 Key Laboratory of Genetics and Germplasm Innovation of Tropical Special Forest Trees and Ornamental Plants, Ministry of Education, Key Laboratory of Germplasm Resources Biology of Tropical Special Ornamental Plants of Hainan Province, College of Forestry, Hainan University, Haikou, Hainan,China; 3 Key Laboratory of Integrated Pest Management on Crops in South China, Ministry of Agriculture, South China Agricultural University, Guangzhou, Guangdong, China; 4 Key Laboratory of Integrated Pest Management on Tropical Crops, Ministry of Agriculture China, Environment and Plant Protection Institute, Chinese Academy of Tropical Agricultural Sciences, Haikou, Hainan, China

**Keywords:** Single-molecule sequencing, RNA-Seq, Transcriptome, Different developmental stage, Differentially expressed genes

## Abstract

**Background:**

Red palm weevil, *Rhynchophorus ferrugineus* Olivier, is one of the most destructive pests harming palm trees. However, genomic resources for *R. ferrugineus* are still lacking, limiting the ability to discover molecular and genetic means of pest control.

**Methods:**

In this study, PacBio Iso-Seq and Illumina RNA-seq were used to generate transcriptome from three developmental stages of *R. ferrugineus* (pupa, 7th-instar larva, adult) to increase the understanding of the life cycle and molecular characteristics of the pest.

**Results:**

Sequencing generated 625,983,256 clean reads, from which 63,801 full-length transcripts were assembled with N50 of 3,547 bp. Expression analyses revealed 8,583 differentially expressed genes (DEGs). Moreover, gene ontology (GO) and Kyoto Encyclopedia of Genes and Genomes (KEGG) enrichment analysis revealed that these DEGs were mainly related to the peroxisome pathway which associated with metabolic pathways, material transportation and organ tissue formation. In summary, this work provides a valuable basis for further research on the growth and development, gene expression and gene prediction, and pest control of *R. ferrugineus*.

## Introduction

Red palm weevil, *Rhynchophorus ferrugineus* Olivier, is one of the most destructive pests that harm palm trees (Ju et al., 2011; Giblin-Davis et al., 2013; Wang et al., 2015). Since *R. ferrugineus* was first reported in 1997 in China, nearly 20,000 coconut palms have been killed, covering more than 10,000 km^2^, and damaging coastal ecosystems in China (Wu et al., 1998; Ju et al., 2011; Shi, Lin & Hou, 2014; Ge et al., 2015). *R. ferrugineus* larvae usually grow in the center of palm trees, feeding on carbohydrate-rich tender tissues and sap and eventually killing the host plant by damaging its meristem (Abe, Hata & Sone, 2009; Butera et al., 2012). This feeding behavior renders detection of the pest difficult until it is already too late, and most traditional methods of pest control, including chemical pesticides, ineffective (Ferry & Gomez, 2002; Faleiro, 2006; Faleiro et al., 2012). Recent studies showed that gut microbiota influenced the development of red palm weevil by regulating nutrient metabolism (Habineza et al., 2019), and understanding the immune stimulating effect of intestinal commensal bacteria on larvae will be beneficial to the formulation of its control strategy (Muhammad et al., 2019). The use of biological control methods to prevent the *R. ferrugineus* (mainly microbes) has achieved certain success, but the application still needs a long time (Mazza et al., 2014). However, as a edible insect, *R. ferrugineus* is rich in nutrients, which makes it has great potential in food industry (Zielińska et al., 2015; Nongonierma & FitzGerald, 2017; Köhler et al., 2019). For example, *R. ferrugineus* larva is a popular edible insect in Papua New Guinea and Thailand (Chung et al., 2002). Recently, high-throughput sequencing technology has been used to obtain transcriptome data from non-model species, providing valuable genomic information even without genome sequences (Morozova, Hirst & Marra, 2009). Transcriptome data provide a useful perspective for elucidating cellular responses, gene function and evolution, and the molecular mechanisms of different biological processes (Hittinger et al., 2010). Therefore, the determination of *R. ferrugineus* genomic information is important, which will effectively help to understand the control strategy of the insect and its potential application as food.

The transcriptome represents all the genes expressed in a cell or a population of cells, and it makes it possible to obtain a biological perspective on cellular processes. RNA-seq transcriptome analysis can effectively identify the temporal and unique gene expression patterns in organisms (Ozsolak & Milos, 2011). RNA-seq was widely used to reveal biological phenomena, gene expression profiles and gene discovery of insects (Ou et al., 2014; Vogel et al., 2014a). Sequence-database can effectively help to understand insect feeding mechanism (Yi et al., 2017), defense (Crava, Brütting & Baldwin, 2016), development process (Ma et al., 2016), and host-pathogen interaction of herbivorous insects (Vogel, Musser & Celorio-Mancera, 2014b). Additionally, studies using transcriptomics-based methods report gene expression analysis of coleopteran pests at different developmental stages (Ma et al., 2016; Li et al., 2017; Yang et al., 2018; Noriega et al., 2019). Meanwhile, RNA interference (RNAi) mechanisms have shown promising results in techniques for controlling coleoptera pests (Vogel, Musser & Celorio-Mancera, 2014b). Sequencing technology can help identifying and selecting RNAi target genes, and is an important tool for insect cellular, genetic and molecular research (Firmino et al., 2013; Zhang et al., 2017). RNAi was considered to be an effective approach of controlling insects and can increase host plant resistance to pests (Baum et al., 2007; Zhu et al., 2010; Niu et al., 2017). Although some researches have been done on the transcriptome of *R. ferrugineus*, transcriptome and gene expression analysis of the pest during developmental stages is still lacking.

Second-generation sequencing is widely used to obtain high transcriptome throughput, but due to the limitation of short read length, the full-length transcripts generated by its assembly are incomplete. Furthermore, the transcripts assembled by second-generation sequencing may be short, incomplete, and lead to incorrect annotations (Au et al., 2013; Li et al., 2018). Compared to short-sequence sequencing, long-sequencing sequencing techniques (such as PacBio) help overcome these limitations without requiring further assembly to provide sequence information for full-length cDNA molecules (Rhoads & Au, 2015). Currently, as single molecule real-time long read sequencing (SMRT) can capture the full-length transcript sequence directly and improve the accuracy of transcriptome characterization and genome annotation, it has been successfully applied to transcriptome analysis in insects, plants, humans, and other animals (Sharon et al., 2013; Larsen, Campbell & Yoder, 2014; Abdel-Ghany et al., 2016; Hartley et al., 2016; Wang et al., 2016; Chen et al., 2017; Zhu et al., 2017; Kawamoto et al., 2019).

In this work, *R. ferrugineus* transcriptome was sequenced and analyzed by Illumina RNA-seq and PacBio Iso-Seq. The transcriptome of pupae, larvae, and adults were compared, then the differentially expressed genes (DEGs) were identified in different functional databases, and the results were analyzed to explore their potential functions. This study will help us to further understand *R. ferrugineus* transcriptome and provide a valuable basis for gene expression and prediction.

## Materials & Methods

### Samples selection

All *R. ferrugineus* samples were collected from the Coconut Research Institute, Chinese Academy of Tropical Agricultural Sciences, Wenchang Hainan, China. *R. ferrugineus* were collected from the field and reared in the laboratory for at least 10 generations. The adults were fed with sugarcane stems in the incubator at 27 ± 1 °C, with the photoperiod of 12 h light/12 h dark, and relative humidity (RH) of 75%, while larvae were artificially reared at 27 ± 1 °C, photoperiod was 24 h dark and 75% RH (Pu et al., 2017). According to our systematic observation of the biological characteristics and morphology, larvae develop in the center of the palm plant and are not easy to be discovered and controlled. With the increase of larval instar, the feeding and exuviating behaviors of the larvae spread from the palm plant center to the periphery. In the development process of larvae, 7th-instar larvae have a longer duration and are more harmful to host trees. As we all know, the more larvae are exposed, the easier to control. Therefore, in this work, a total of 12 healthy and whole individuals were collected from the reared population for sequencing, including three 7th-instar larvae, three pupae, three female adults, and three male adults. Samples were flash-frozen in liquid nitrogen and stored at −80 °C.

### RNA extraction and sequencing

The RNeasy Kit (Qiagen, Valencia, CA, USA) was used to extract total RNA from each whole individual sample of *R. ferrugineus*. The integrity and concentration of RNA were measured using Agilent 2100 (Agilent Technologies, USA) and Qubit^®^ RNA Assay Kit in the Qubit^®^ 2.0 Fluorometer (Life Technologies, CA, USA), respectively. The purity of RNA (OD 260/280) was tested using Nanodrop (NanoDrop products, USA), while the contamination and degradation of RNA was analyzed using 1% agarose gels. For PacBio Iso-Seq, total RNA from 12 samples were mixed together and 2 µg RNA was added to per sample. On the Pacbio platform (Pacific Biosciences, CA, USA), 3 µg RNA from total mixed RNA was sequenced according to the manufacturer’s instructions. Then, according to the content of Pacific Biosciences (PN 100-092-800-03), the BluePippin size selection system protocol and the Clontech SMARTer PCR cDNA synthesis kit were used to prepare the Iso-Seq library. For Illumina RNA-Seq, the library was prepared by NEBNext^®^ Ultra™ RNA Library Prep Kit. Briefly, the enriched mRNA was purified using Oligo (dT) magnetic beads. Subsequently, the mRNA was processed into short fragments by fragmentation buffer, and a strand of cDNA was synthesized by random hexamers using the mRNA as the template. Then AMPure XP beads were used to purify the two-strand cDNA synthesized by buffer, DNA polymerase I and dNTPs. Finally, AMPure XP beads were used to select 250∼300 bp fragments, and the final cDNA library was obtained through PCR enrichment. Qubit^®^2.0 Flurometer (Life Technologies,CA, USA) was used to perform preliminary quantification of the constructed library, and the library was diluted to 1 ng / µL. Then Agilent 2100 was adopted to detect the insert size length of the library, and the Q-PCR kit (TaKaRa, Dalian, China) was used to accurately quantify the effective concentration of the library (effective library concentration>2 nM) to ensure the quality of the library. Twelve libraries were prepared for Illumina sequencing. The Novaseq 6000 sequencing platform was used to sequence each sample of *R. Ferrugineus* with 1.5 µg RNA. The short-read sequencing data and the full-length transcriptome reference sequence of *R. ferrugineus* can be obtained in NCBI under accession ID PRJNA598560.

### Data processing

SMRTlink6.0 software was applied to process the sequence data (Chin et al., 2013). The cyclic consensus sequence (CCS) was generated from the subread BAM files (parameters: min_length 50, max_drop_fraction 0.8, no_polish TRUE, min_zscore-9999.0, min_passes2, min_predicted_accuracy 0.8, max_length 15000). CCS.BAM files were output, which were then classfied into full-length and non-full-length reads using pbclassify.py, ignorepolyA false, minSeqLength 200. The two ends of the sequence contain both 3′ primer and 5′ primer, and the sequence containing the poly (A) tail before the 3′ primer is full-length read, otherwise, it is non-full-length read. The generated non-full-length and full-length fasta data files were processed by isoform-level clustering, and then Quiver (parameters: hq_quiver_min_accuracy 0.99, bin_by_primer false, bin_size_kb 1, qv_trim_5p 100, qv_trim_3p 30) was used for arrow polishing (Pacific Biosciences, 2014). The clean data were collected from Illumina Novaseq 6000 sequencing platform and processed as usual. In this step, clean reads were obtained by removing reads containing adapter, low quality reads and reads containing ploy-N from raw data. At the same time, phred score (Q20, Q30), error rate, GC-content and sequence duplication level of the clean data were calculated. In order to further improve the accuracy of sequencing, LoRDEC software was conducted to correct the PacBio Iso-Seq data using the Illumina RNA-seq data (Salmela & Rivals, 2014). The calibration process was performed as the following steps: firstly, LoRDEC was adopted to obtain second-generation short-reads and to construct DBG (DE Bruijn Graph) graphs. Then, each third-generation long-reads were read in turn by LoRDEC to determine whether the third-generation data was supported by the second-generation data in the constructed DBG graphs. Finally, the data that was not supported by the second-generation data was corrected, and then the corrected sequence was output. The corrected sequence was removed by CD-HIT (parameters: -c 0.95 -T 6 -G 0 -aL 0.00 -aS 0.99) software for any redundancy, and the transcript sequence for subsequent analysis was obtained (Fu et al., 2012).

### Quantification of gene expression levels

Full-length transcripts from the PacBio data was adopted as a reference sequence (ref), then using Bowtie2 software to map the clean reads of each sample from Illumina sequencing to ref (parameters: -end-to-end -no-mixed -no-dis-cordant -gbar 1000 -k 200) (Langmead & Salzberg, 2012). The expression level of each transcript for each sample was calculated and normalized into TPM (Transcripts Per Million) values by RSEM software (Li & Dewey, 2011), and classfied into five categories including very low, low, moderate, high and very high with the TPM values of 0-0.1, 0.1-1, 1-5, 5-15, 15-60, >60, respectively.

### Differentially expressed genes

Prior to differential gene expression analysis, for each sequenced library, the read counts were adjusted by edgeR program package through one scaling normalized factor. Then, calculate the probability of statistical hypothesis testing according to the negative binomial distribution model; finally, the multiple hypothesis test correction was performed to obtain the false discovery rate (FDR). The FDR is a statistical measure used to determine the threshold for *P*-values in multiple tests which accounts for the proportion of false positives (Benjamini & Yekutieli, 2001). The DESeq (Anders & Huber, 2010) R package (1.10.1) was used to analyze the differential expression between the two groups. DESeq can provide routine statistics to determine differences in digital gene expression data using a model based on the negative binomial distribution. The *P*-value of the result was adjusted using the method of Benjamini and Hochberg to control the false discovery rate. Genes with an adjusted *P*-value <0.05& —log2 (foldchange)—>0 found by DESeq were assigned as differentially expressed. Transcription factors (TFs) among the DEGs were predicted using iTAK software (v1.2). In this work, Hierarchical cluster (H-cluster) (Murtagh, 1983), K-means (Selim & Ismail, 1984) and Self-organizing Map (SOM) (Kohonen, 1988) were adopted to cluster the relative expression levels of differential genes. H-cluster was used to calculate the Euclidean distance between samples, and then the most similar variables were clustered step by step to achieve sample clustering. K-means is a method of cluster analysis, which groups observations by minimizing the Euclidean distance between them. The basic self-organizing system is a one- or two- dimensional array of processing units resembling a network of threshold-logic units, and characterized by short-range lateral feedback between neighbouring units.

### GO and KEGG pathway enrichment analysis

Gene Ontology (GO) functional classification analysis of DEGs were performed by the GOseq R package (Young et al., 2010). GO terms (http://www.geneontology.org/) with corrected *P*-value<0.05 were considered significantly enriched by DEGs. All the DEGs were mapped to the GO database, and then the significantly enriched terms compared to the entire genome background were identified. KOBAS software (Xie et al., 2011) (http://kobas.cbi.pku.edu.cn) was implemented to test the statistical enrichment of DEGs in KEGG (Kyoto Encyclopedia of Genes and Genomes) pathways. Hypergeometric test was used for KEGG enrichment analysis to identify pathways in which the DEGs were significantly enriched relative to all the annotated genes. KEGG pathways with corrected *P*-value<0.05 were determined as significantly items.

## Results

### Summary of Illumina Novaseq 6000 and PacBio SMRT transcriptome sequencing data

A total of 12 samples (three male adults, three female adults, three pupae and three larvae) were sequenced using Illumina Novaseq 6000, and each sample generated > 6 GB of transcriptome data. Illumina Novaseq 6000 platform generated 642,179,304 raw reads and 625,983,256 clean reads (97.48%, 93.91Gb) with an arithmetic average Q30 value of 93.24%, an arithmetic average Q20 value of 97.70% and an arithmetic average GC content of 37.71% (Table 1). The PacBio sequencing platform obtained a reference transcript containing 63,801 full-length transcripts (N50 length of 3,547 bp and N90 length of 1,921 bp) after clustering with CD-HIT-EST, totaling 417,431,883 bp. Subsequently, clean reads of each Illumina sequenced sample were mapped to full-length transcripts. In terms of the arithmetic average, 88.32%, 87.55%, 89.35%, and 86.98% of short reads were successfully mapped for larvae, pupae, female adults, and male adults, respectively (Table 1).

**Table 1:** Summary of quality of RNA-Seq original sequencing data. Raw reads column lists each read in the FASTQ format file by four lines and a unit, and counts the number of sequences in each file. Clean reads column lists the filtered sequencing data of raw reads, and the subsequent bioinformation analysis is based on clean reads. Clean bases column lists the number of clean reads multiply the length, and G represents the data size of clean reads. Q20 column listed the percentages of bases with phred quality score greater than 20. Q30 column listed the percentages of bases with phred quality score greater than 20. GC content column lists the total number of bases G and C as a percentage of the total number of bases. Total mapped column lists sequence count statistics that can be mapped to a reference sequence.

**Sample name**	**Raw reads**	**Clean reads**	**Cleanbases**	**Q20(%)**	**Q30(%)**	**GC content(%)**	**Total mapped(%)**
larva_1	45,921,768	45,216,478	6.78G	97.84	93.51	39.10	89.14%
larva_2	45,488,706	44,719,458	6.71G	98.15	94.41	39.17	87.93%
larva_3	46,968,766	43,815,018	6.57G	98.20	94.52	39.61	87.88%
pupa_1	51,547,360	50,918,894	7.64G	97.82	93.42	40.09	89.11%
pupa_2	49,213,402	48,731,238	7.31G	97.85	93.58	40.66	86.42%
pupa_3	47,589,126	45,987,494	6.9G	97.88	93.68	41.13	87.12%
female_1	65,486,770	64,749,568	9.71G	97.47	92.46	32.48	91.53%
female_2	54,512,618	51,662,048	7.75G	97.20	92.14	34.17	87.69%
female_3	49,813,910	48,435,084	7.27G	97.31	92.31	34.11	88.84%
male_1	62,939,496	60,971,604	9.15G	97.51	92.88	38.99	84.75%
male_2	69,968,690	69,071,824	10.36G	97.53	92.94	38.33	85.44%
male_3	52,728,692	51,704,548	7.76G	97.66	93.00	34.71	90.75%

### Analysis of gene expression levels

**Figure 1:**
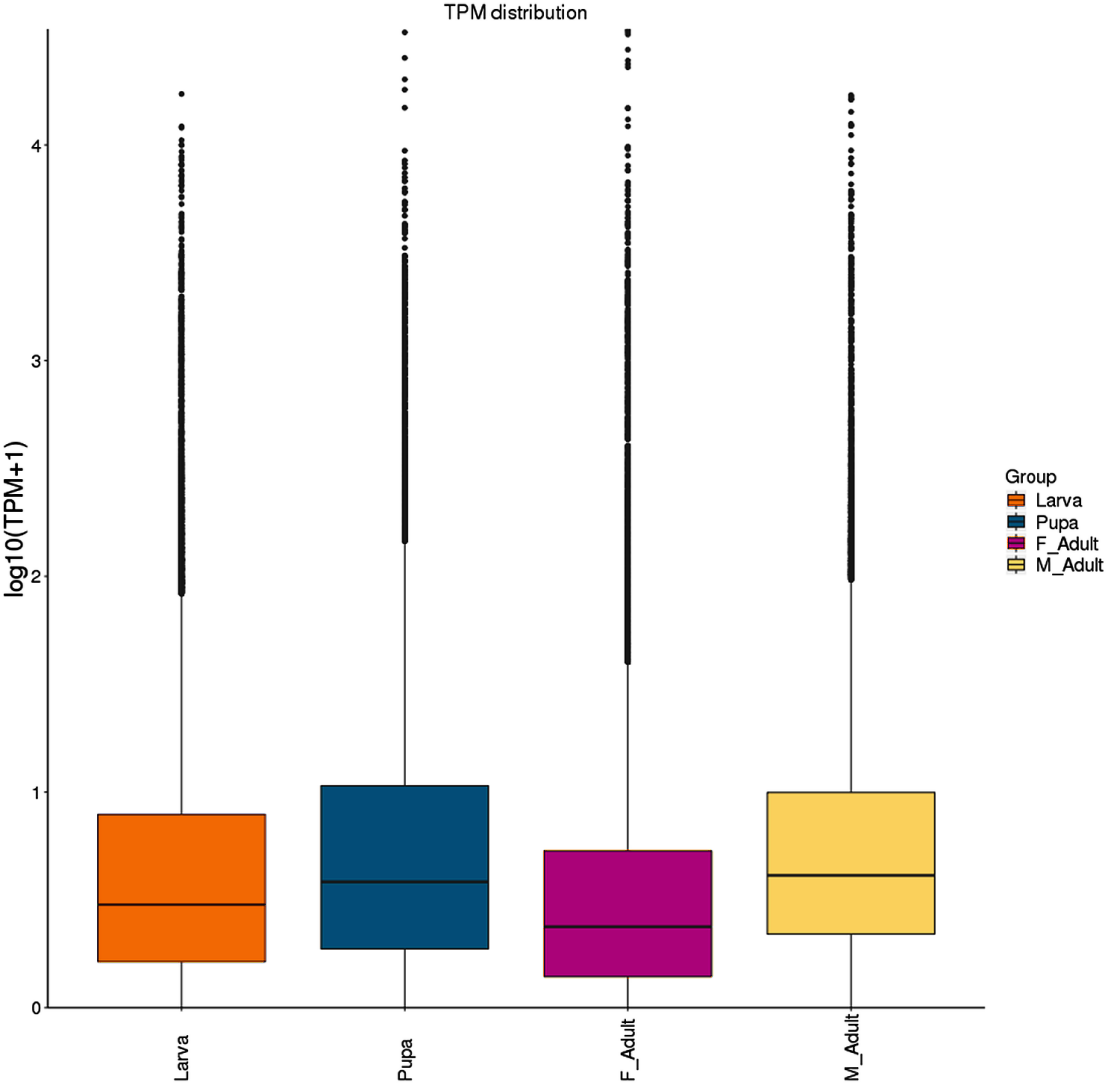
Boxplot of the log transformed TPM expression values of different developmental stages. The solid horizontal line represents the median, and the box encompasses the lower and upper quartiles as well as the maximum and minimum values. TPM was Transcripts Per Million, F_Adult_ was female adults, M_Adult_ was male adults. The abbreviations TPM, F_Adult_ and M_Adult_ in Figs. 2–6 have the same meaning as here.

**Figure 2:**
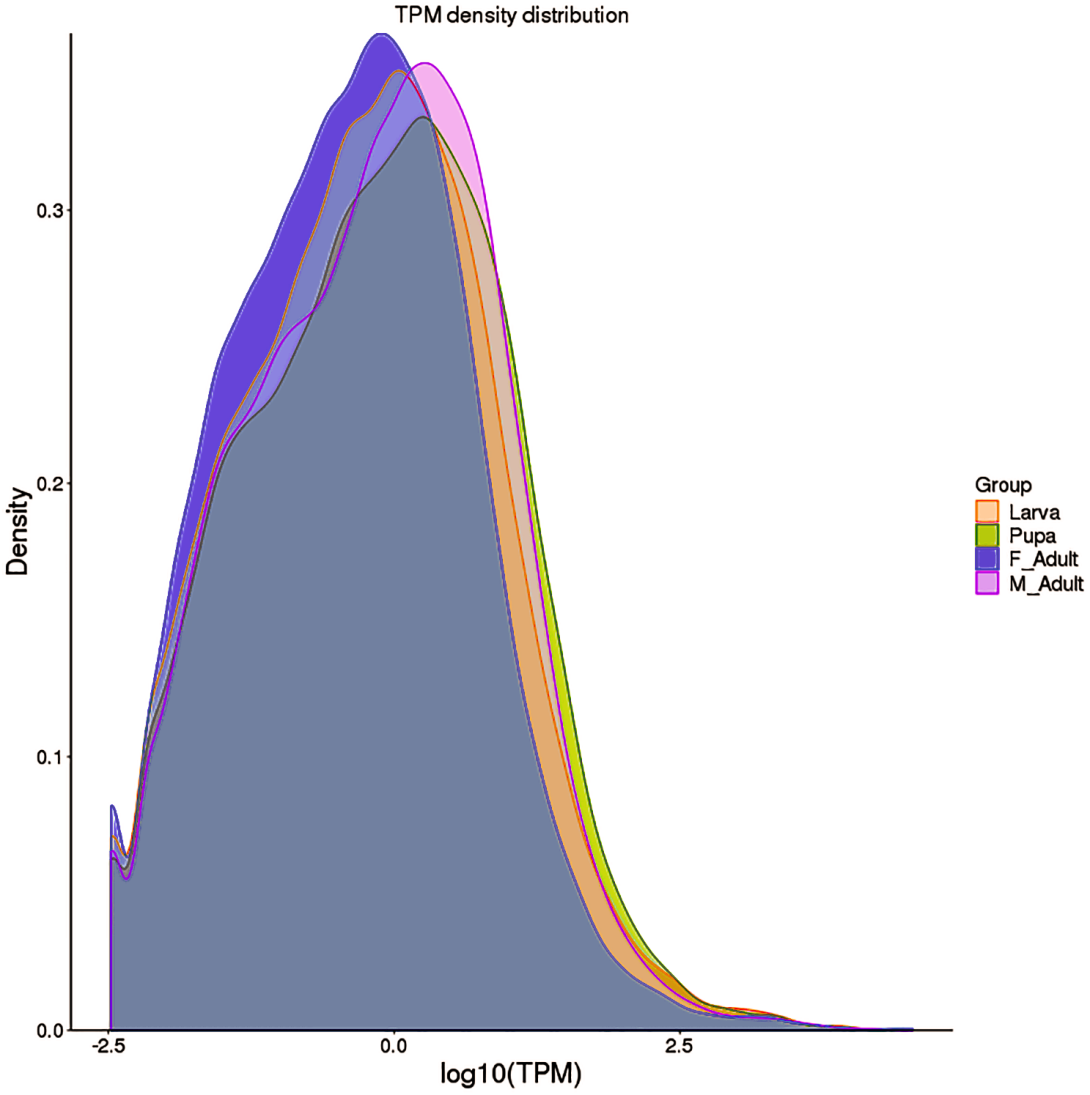
Gene expression density map (comparison of gene expression levels). The abscissa is the log_10_ (TPM) value of the gene, the higher the value, the higher the gene expression; the ordinate is the density corresponding to log_10_ (TPM), and different colors represent different samples.

TPM box plots of gene expression values of different developmental stages indicated that the overall data volume of each sample gene expression was substantially consistent. The median value was larger and the overall expression level of genes was relatively high in pupae and male adults (Fig. 1). The main distribution range of TPM value was 0–5 (Fig. S1). Meanwhile, the overall gene expression levels of *R. ferrugineus* at different developmental stages were not significantly different (Fig. 2 and Table S1).

### Identification of Differentially Expressed Genes (DEGs)

**Figure 3:**
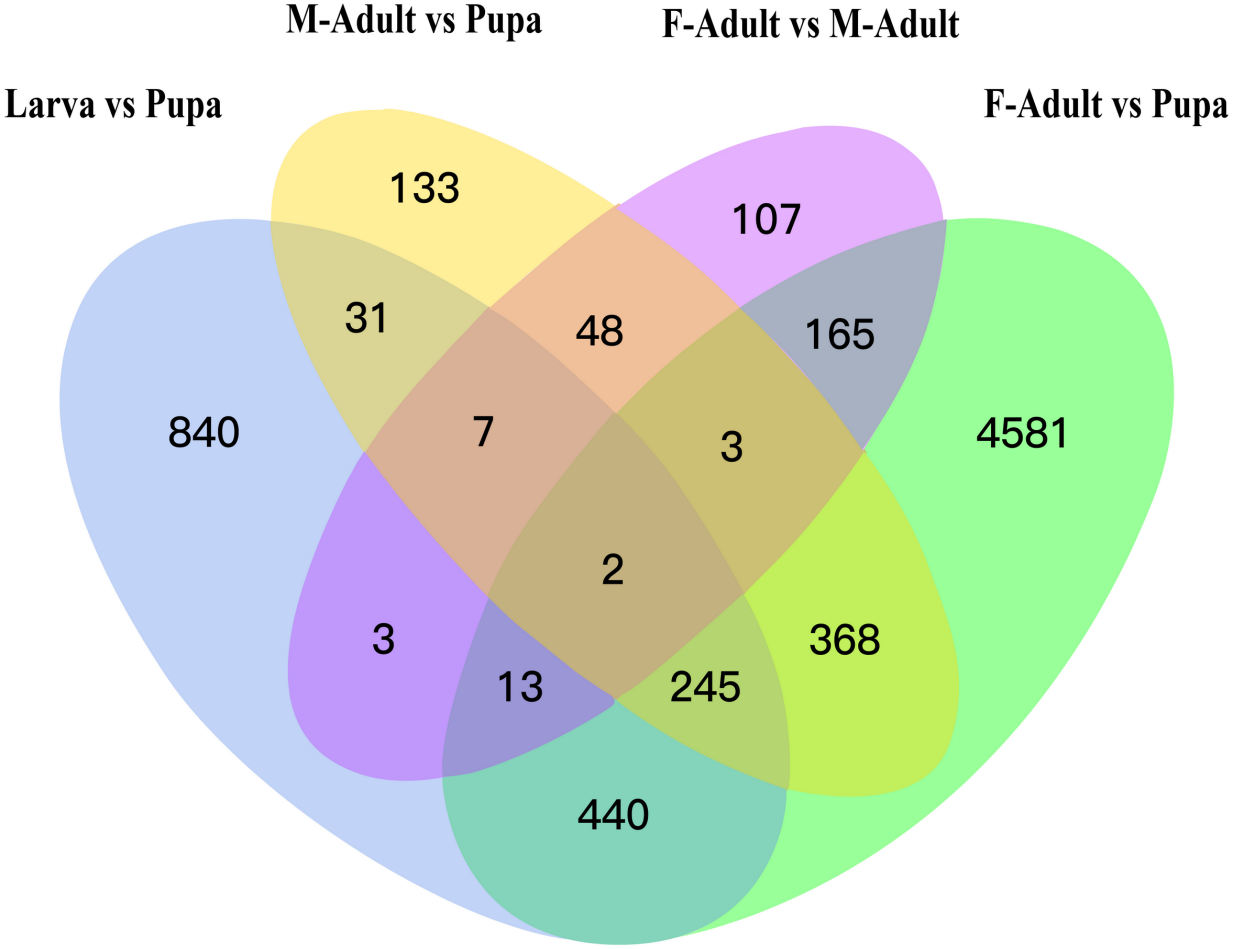
Venn diagram for DEGs at different development stages of *R. ferrugineus*.

To identify DEGs during the development of *R. ferrugineus*, volcano maps were implemented to show the overall distribution of DEGs at different developmental stages. A total of 8,583 DEGs were identified, of which 1,581 (1,054 up- and 527 down-regulated), 837 (421 up- and 416 down- regulated), 5,817 (3,328 up- and 2,489 down- regulated), and 348 (172 up- and 176 down-regulated) were found between larvae and pupae, pupae and male adults, pupa and female adults, and female adults and male adults, respectively (Fig. S2). Meanwhile, differential genes were clustered by log2 (ratios) of relative expression level by H-cluster, K-means and SOM methods. Different clustering algorithms divided the DEGs into several clusters. The grey line in the clustering result graph represented the relative expression of genes in a cluster at different development stages (based on the expression level of the first sample, as shown in the red line), while the blue line represented the average of the relative expression of all genes in the cluster at different development stages (Figs. S3, S4 and S5). The results demonstrated that genes in the same cluster have similar expression levels at different developmental stages. Then, in order to visually show the number of common and unique DEGs among the groups, pairwise comparisons were made among the number of DEGs in the different development stages, and a Venn diagram was drawn. The results indicated that the number of DEGs between pupa and larva, pupa and female adults, pupa and male adults, and female adults and male adults were 1,581, 5,817, 837, and 348, respectively (Fig. 3).

### GO and KEGG enrichment analysis of DEGs

**Figure 4:**
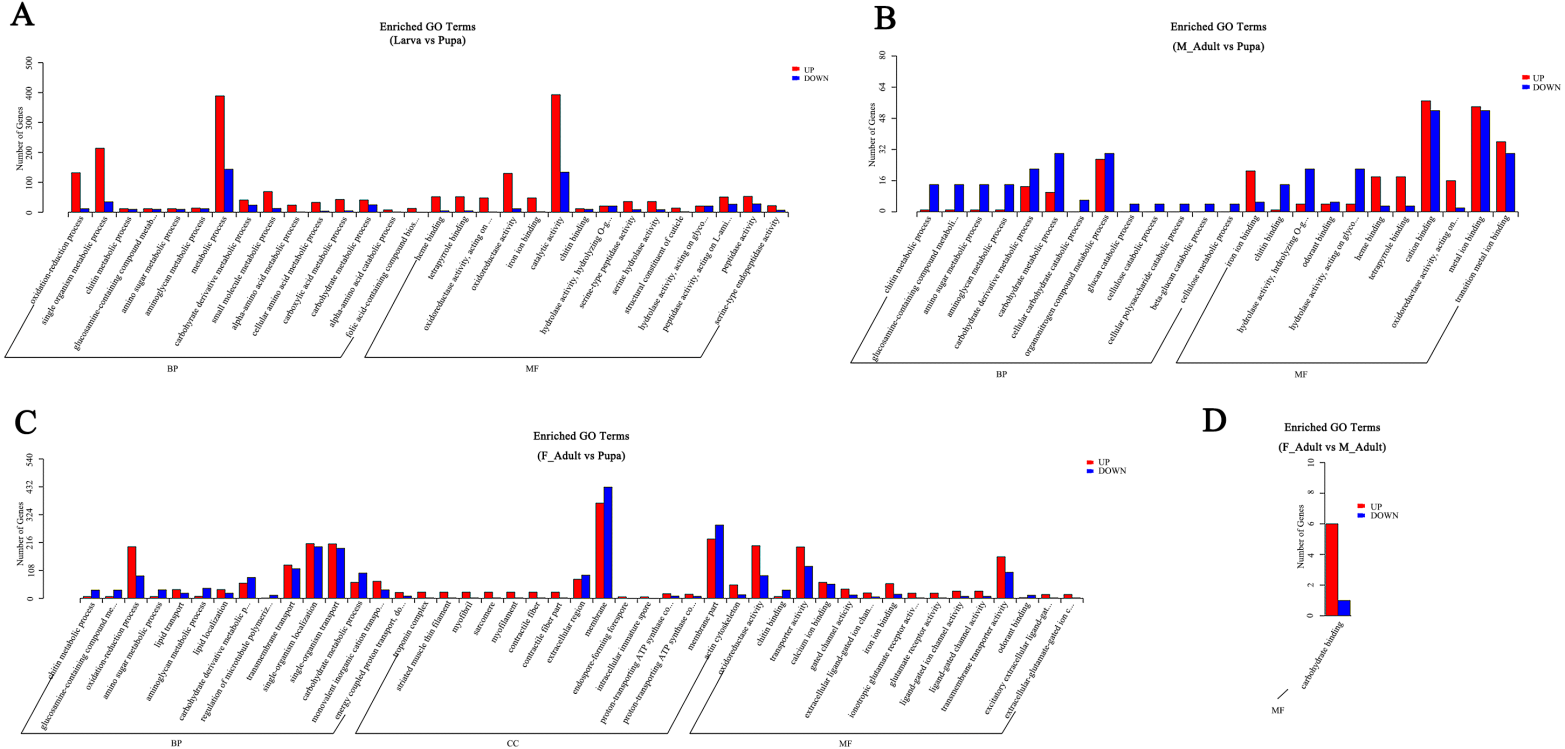
GO enrichment analysis of DEGs that compared among the different developmental groups. (A) GO functional enrichment analysis of DEGs in Larva vs Pupa. (B) GO functional enrichment analysis of DEGs in Male Adults vs Pupa. (C) GO functional enrichment analysis of DEGs in Female Adults vs Pupa. (D) GO functional enrichment analysis of DEGs in Male Adults vs Female Adults. Blue, downregulated. Red, upregulated. BP, biological process. CC, cellular component. MF, molecular function.

**Figure 5:**
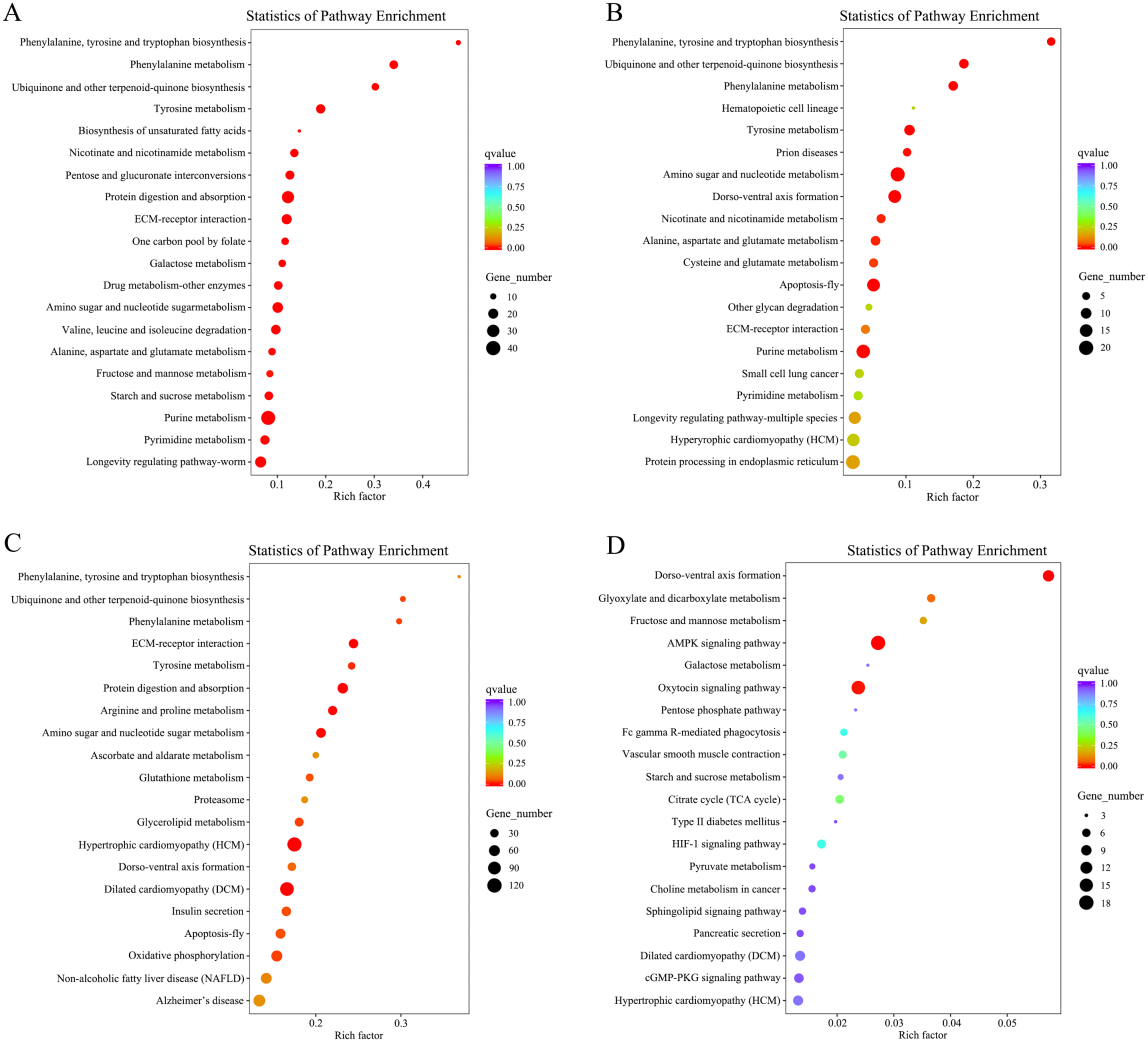
KEGG enrichment analysis of DEGs that compared among the different developmental groups. (A) Scatter plots of KEGG enrichment results in Larva vs Pupa. (B) Scatter plots of KEGG enrichment results in Female Adults vs Pupa. (C) Scatter plots of KEGG enrichment results in Male Adults vs Pupa. (D) Scatter plots of KEGG enrichment results in Male Adults vs Female Adults. Rich factor represents the ratio of the number of genes located in the pathway among differential genes to the total number of genes located in the pathway among all annotated genes. qvalue represents the p-value after multiple hypothesis testing and correction, and the value range of qvalue is [0,1]. The closer qvalue is to zero, the more significant the enrichment.

Based on the DEGs, enrichment analysis of GO and KEGG were performed to associate DEGs with biological pathways and functional groups. Between larvae and pupa, the DEGs were mainly enriched in the biological processes and molecular functions of 30 GO terms, several of which were significantly enriched in terms related to metabolic pathways, including: single-organism metabolic process, metabolic process, oxidoreductase activity, catalytic activity and amino acid metabolism (Fig. 4A and Table S2). Meanwhile, KEGG enrichment analysis showed that 250 pathways were enriched with corrected *P*-value < 0.05, the significantly enriched KEGG pathways were primarily related to energy supply (amino sugar and nucleotide sugar metabolism, fructose and mannose metabolism, protein digestion and absorption, galactose metabolism), the amino acid metabolism (phenylalanine metabolism, tyrosine metabolism, nicotinate and nicotinamide metabolism) (Fig. 5A and Table S3).

Regarding the comparison between pupal and adult stages, among the encoded functional groups, the significantly enriched KEGG pathways were primarily involved in amino acid synthesis and metabolism, glucose metabolism, and tissue and organ development. For example, phenylalanine, tyrosine and tryptophan biosynthesis, phenylalanine metabolism, amino sugar and nucleotide sugar metabolism, amino sugar and nucleotide sugar metabolism, other glycan degradation, dorso-ventral axis formation, protein processing in endoplasmic reticulum, hematopoietic cell lineage (Figs. 5B and 5C, and Table S4). The significantly enriched GO terms were mainly involved in organ tissue formation (membrane, membrane part and actin cytoskeleton), material transportation (transmembrane transport, single-organism transport, transporter activity and transmembrane transporter activity) and molecular function (iron ion binding, heme binding, cation binding, metal ion binding and transition metal ion binding) (Figs. 4B and 4C, and Table S5). When differential genes expression was analyzed between the female adults and male adults, significantly enriched KEGG pathways (AMPK signaling pathway and oxytocin signaling pathway) and GO terms (carbohydrate binding) were mainly associated with signal transduction and metabolism (Figs. 4D and 5D, and Table S6).

### DEGS analysis of TFs and Long noncoding RNAs (LncRNAs)

**Figure 6:**
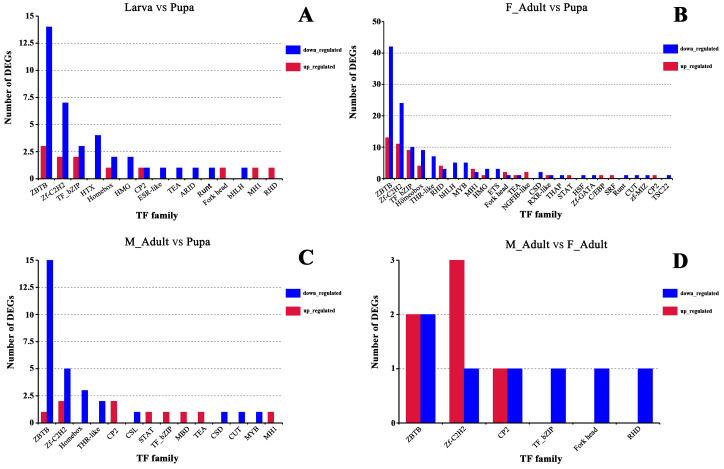
Number of genes up-regulated and down-regulated in the TF family in different comparison groups. (A) TFs analysis between Larva vs Pupa. (B) TFs analysis between Female adults vs Pupa. (C) TFs analysis between Male adults vs Pupa. (D) TFs analysis between Male adults vs Female adults. Blue, downregulated. Red, upregulated.

TFs play an important regulatory role in animal growth and development as well as in insect immunity and other aspects. Therefore, this study investigated the dynamics of TFs expression during *R. ferrugineus* development. The comparison among these different development stages found that 50 (12 up- and 38 down-regulated), 180 (55 up- and 125 down-regulated), 39 (10 up- and 29 down-regulated) and 13 (6 up- and 7 down-regulated) TFs had significantly differential expression in larvae vs pupae, pupae vs female adults, pupae vs male dults, female adults vs male adults, respectively. Besides, larvae and pupa up-regulated TFs mainly comprised the following TFs families: zf-C2H2, ZBTB, TF_bZIP. Down-regulated differential TFs mainly comprised the following TFs families, including zf-C2H2, ZBTB, TF_bZIP, HTH (Fig. 6A). For female adults vs pupae, male adults vs pupae, the main families of up-regulated TFs were zf-C2H2, Homeobox and ZBTB, and the main families of down-regulated TFs were zf-C2H2, ZBTB and TF_bZIP (Figs. 6B and 6C). Regarding the comparison of the female and male stages, TFs were up-regulated in the zf-C2H2, ZBTB and CP2 families. Down-regulation of TFs were mainly in the zf-C2H2 and ZBTB families (Fig. 6D). Meanwhile, transcripts predicted to be LncRNAs were screened from the DEGs combinations at different developmental stages, and up- and down-regulated LncRNAs in different comparison combinations were obtained. A total of 81 (including 56 up- and 25 down-regulated DEGs), 477 (including 416 up- and 61 down-regulated DEGs), 44 (including 25 up- and 19 down-regulated DEGs), and 13 probable LncRNAs (including 6 up- and 7 down-regulated DEGs) were identified from different combinations of larvae vs pupae, pupae vs female adults, pupae vs male adults, female dults vs male adults, respectively (Fig. S6).

## Discussion

Native to Southeast and South Asia, *R. ferrugineus* invaded the Arab states in the Persian Gulf in the mid-1980s and is now found all over the world (Bozbuga & Hazir, 2008). The adults are large (body length of about three cm), reddish brown with sturdy wings, and are adapted for long-distance flight (Salama, Zaki & Abdel-Razek, 2009). The weevil’s trunk-boring life style makes them hard to control (Salama, Zaki & Abdel-Razek, 2009). Although multiple researches have been done to find new ways to control *R. ferrugineus*, including chemical agents (Pugliese et al., 2017; Wakil et al., 2018), biological agents (Zulkifli, Zakeri & Azmi, 2018), and intestinal microbes (Habineza et al., 2019; Muhammad et al., 2019), the results are not effective. There are few studies on the molecular characteristics of *R. ferrugineus*, including transcriptome, genome structure and proteome (Muhammad et al., 2019). Transcriptomics provides complementary data and gene expression data for available genomes for organisms at specific conditions or at different stages of development. Transcriptome sequencing has become an important tool for studying gene expression and regulation. Through the functional description of a large number of genetic data sets and the analysis of the expression of differential genes, valuable information can be provided for insects control strategies. Recently, transcriptome of developmental stages of various coleopteran insects have been sequenced, including *Nicrophorus orbicollis* (Won et al., 2018)*, Hypothenemus hampei* (Noriega et al., 2019) and *Batocera horsfieldi* (Yang et al., 2018). In addition, due to the lack of reference genomes, transcriptome sequencing places higher requirements on sequencing tools (Martin & Wang, 2011). Third-generation sequencing can directly obtain complete transcripts, overcoming the problems of short transcript assembling and incomplete information of non- reference genome species, so it reflects the information of transcriptome sequencing species more realistically, making it more widely used in transcriptome analysis (Rhoads & Au, 2015). In this work, transcriptome of *R. ferrugineus* was reported using RNA-seq and PacBio Iso-Seq, and 625,983,256 clean reads and 63,801 full-length transcripts with an average length of 2,964 bp were obtained respectively. The average length of the acquired transcriptome data in the *R. ferrugineus* was higher than that of other members of Curculionidae, such as *Anthonomus grandis* (average length 237 bp) (Salvador et al., 2014), *Eucryptorrhynchus chinensis* (average length 360 bp) (Liu & Wen, 2016), *Tessaratoma papillosa* (average length 1,095 bp) (Wu et al., 2017) and *Hypothenemus hampei* (average length 1,609.92 bp) (Noriega et al., 2019). Here, the sequenced full-length transcriptome of *R. ferrugineus* provided a clearer picture of molecular changes underlying development.

DEG analysis revealed 8,583 loci involved in metabolic pathways, material transportation, and organ tissue formation. Many physiological processes change between the larval and pupa stages. To pupate successfully, the last larval instar needs to reach a critical weight, which allows it to pupate even if it has not eaten any more food (Nijhout & Williams, 1974; Mirth, Truman & Riddiford, 2005; Tobler & Nijhout, 2010; Keshan, Thounaojam & Kh, 2015). In this work, the major differentially expressed genes in the development of larvae and pupa were enriched in KEGG pathways: phenylalanine metabolism , tyrosine metabolism , phenylalanine, tyrosine and tryptophan biosynthesis , ubiquinone and other terpenoid-quinone biosynthesis. Furthermore, studies on *Cylas formicarius* showed that the most important KEGG pathways of DEGs during development were pancreatic secretion, lysosome and metabolic pathways, and these pathways may play important roles in different stages of insect development (Ma et al., 2016). At the same time, through the KEGG analysis of *Nicrophorus orbicollis* and *Hypothenemus hampei* at different development stages, the resluts showed that functional pathways are mainly related to carbohydrate metabolism, immune system, signal transduction (Won et al., 2018; Noriega et al., 2019). In this work, the data supported the biological differences between the larval and the pupal development stages of *R. ferrugineus*, which were mainly enriched in the synthesis and metabolism of substances. In this process, the larvae acquire energy through a large amount of food intake, and prepares for pupation by synthesizing, digesting, and metabolizing the substance. Our data also provided the observation and research conclusions that larvae bear the common digestive function of the population. For example, when collecting food, adult workers adjust their harvesting strategy according to the nutritional needs of the individuals in the colony, and the larvae will consume the protein of the entire colony to satisfy developmental needs (Dussutour & Simpson, 2009). Moreover, in this work, the DEGs of larvae and pupae were also enriched in serine hydrolase activity and peptidase activity pathways. Some related studies have shown that the larval stage belongs to the feeding stage, so the expression of genes related to digestion and metabolism is higher (Ma et al., 2016; Tang et al., 2017). Insect genes can respond to targeted digestive enzyme inhibitors in a timely manner and evolve rapidly, which means that accurate recognition and identification of these genes will facilitate the formulation of pest control strategies (Zhu-Salzman & Zeng, 2015). However, the function of these genes in *R. ferrugineus* needs further studies to verify.

Expression of storage proteins in the larval stage is a way to store nutrients and energy (Wheeler & Martinez, 1995). These storage proteins can be degraded during the pupa stage to provide energy for development (Chen, 2015). The results of *R. ferrugineus* indicated that 5,817 and 837 DEGs were expressed in pupae and female adults, male adults and pupae respectively. GO enrichment analysis showed that the DEGs between the pupal and adult stages were significantly enriched as cation binding , metal ion binding , membrane and membrane part terms. At the same time, KEGG enrichment analysis declared that the main enrichment pathways of DEGs were phenylalanine metabolism amino sugar and nucleotide sugar metabolism, dorso-ventral axis formation, phenylalanine, tyrosine and tryptophan biosynthesis, and ECM-receptor interaction. The extracellular matrix (ECM) consists of complex structural and functional macromolecules that provide structural support for organs and tissues and provide structural support for cell layers in the form of cell membranes (Holt & Bullock, 2009). The results of *R. ferrugineus* demonstrated that GO enrichment was a significant item of membranes. Specific interactions between cells and ECM control cellular activities such as proliferation, differentiation, migration, apoptosis and adhesion (Lanza, Langer & Chick, 1997; Nelson & Bissell, 2006). Thus, this pathway may play an important role in the development of *R. ferrugineus* from pupa to adult. In addition, in the comparison between pupa and adult stage, DEGs was significantly enriched in the pathway of tissue and organ formation. For example, the enrichment analysis of GO terms suggests that the DEGs were mainly related to various developmental processes, including organelle assembly, developmental maturity, cell division and cellular component biogenesis. Meanwhile, the DEGs were also enriched in GO terms related to actin filament organization. The rearrangement and stability of the cytoskeleton has become a relevant topic for insects to adapt to low temperature environments (Kim et al., 2006; Des Marteaux, Štětina & Koštál, 2018) and cold injury repair (Kayukawa & Ishikawa, 2009; Teets et al., 2012). Especially in the defense of polymeric actin, it is the main research field. The transcriptome data obtained in this work will provide more information for us in understanding the molecular mechanisms of *R. ferrugineus*, especially in the pupa-to-adult stage.

In this work, arachidonic acid metabolism, glycerophospholipid metabolism, linoleic acid metabolism and biosynthesis of unsaturated fatty acids, which are related to fatty acid metabolism, were identified in KEGG enrichment pathways. Many kinds of fatty acid are contained in *R. ferrugineus*, including palmitic acid, oleic acid, linoleic acid, *α*-linolenic acid, arachidonic acid, lacric acid, myristic acid, etc. Palmitic (49.4–53.3%) and oleic (42.2–46.9%) acids are the major fatty acids in *R. ferrugineus* larvae (Khanittha, Manat & Worawan, 2020). Phospholipid synthesis requires a variety of cofactors and enzymes, which are important components of life, and some phospholipids are essential nutrients (Borrelli & Trono, 2015). In general, the level of phospholipids in insects is maintained at 0.4% to 3.3%, which plays an important role in the production of industrial lecithin (phospholipid mixtures) (Paul et al., 2016). The phospholipids extracted from insects are powerful and widely used in food additives, cosmetics, medicine and other related industries (Duan, 2005). Related research shows that *R. ferrugineus* is rich in phospholipids (2.6–9.3 g/100 g lipid) (Khanittha, Manat & Worawan, 2020). Moreover, the average protein content of edible insects is 10%–25% of fresh weight or 35%–60% of dry weight (Melo et al., 2011; Schlüter et al., 2016), even higher than the protein content of grains, lentils and soybeans (Bukkens, 1997). In fact, the dietary protein derived from insects is close to 50%, which is more market value than other proteins (Dobermann, Swift & Field, 2017). *R. ferrugineus* is also rich in protein (18.0–28.5%, dry weight) (Khanittha, Manat & Worawan, 2020). Besides, the red palm weevil is rich in macro- (potassium, phosphorus, magnesium, sodium, and calcium) and micro- (zinc, manganese, iron, and copper) elements. The explorations of the nutritional composition of *R. ferrugineus* will be beneficial to the development of the species as food. More importantly, the genetic variation of different developmental stages enriches the genetic profiles of *R. ferrugineus*. This has the potential to help identify RNAi targets that will contribute to insect control (Upadhyay et al., 2011; Shukla et al., 2016). However, further validation will be needed in future studies to elucidate the functions of these DEGs.

The *limpet* transcription factors are related to insect immune response and affect the survival of fungus-free insects, such as protecting *Triatoma infestans* from being infected by *Beauveria bassiana* (Jin et al., 2008; Altincicek, Knorr & Vilcinskas, 2008; Mannino, Paixão & Pedrini, 2019). It is possible that some TFs have evolved to take in different metabolic processes and to present multiple or divergent functions even having a similar nucleotide sequence (Chen & Rajewsky, 2007). DEGS analysis of TFs showed that the mainly TFs families of different development *R. ferrugineus* combinations were ZBTB, zf-C2H2, TF_bZIP, etc. Various transcription factors are included in the ZBTB family, and some ZBTB proteins are key factors in regulating developmental events and lymphoid cell function (Zhu et al., 2018). Members of the CP2 / Grh (Grainyhead) family are found in diverse taxa, ranging from fungi to animals, such as *Drosophila*, the first member of the CP2/Grh (Nüsslein-Volhard, Wieschaus & Kluding, 1984; Bray & Kafatos, 1991). CP2/ Grh transcription factor is extremely stable in multicellular organisms and is a key regulator of organ development and epithelial differentiation (Ming et al., 2018). With the continuous deepening of the research on the function of lncRNAs, more and more insect lncRNAs have been discovered, such as *Nasonia vitripennis*, *Nilaparvata lugens*, *Plutella xylostella*, which provide a research basis for insect resistance, growth and development (Zhu, Liang & Gao, 2016). In order to clarify the function of TFs and lncRNAs in insects development, it is necessary to further study many unknown sequences involved in developmental function and immune response in the future.

## Conclusions

In this work, *R. ferrugineus* transcriptome data of three different developmental stages were obtained successfully by mapping short RNA-seq short reads to the full-length transcripts. The DEGs data will provide information on the identification of genes involved in the development of *R. ferrugineus* and supply molecular information on its application as a potential food resource. The transcriptome data analyses of genes related to insect development will be helpful for pest control.

##  Supplemental Information

10.7717/peerj.10223/supp-1 Supplemental Information 1 Supplemental Figures and Tables

## Additional Information and Declarations

### Data Availability

The following information was supplied regarding data availability: Illumina RNA-seq sequencing data generated from R. *ferrugineus* are available at NCBI: PRJNA598560.
