# Supplementary material for: Transcriptome and gene expression analysis of Rhynchophorus ferrugineus (Coleoptera: Curculionidae) during developmental stages
Source: PeerJ. 2020 Nov 2;8:e10223. doi: 10.7717/peerj.10223 (PMC7643551; doi:10.7717/peerj.10223)
Supplement: Supplemental Information 1 [file peerj-08-10223-s001.zip › Supplemental Files/Supplementary Figures/Supplementary material Figure legends.docx]

**Supplementary material Figure legends**

Supplementary material Figure S1: Statistical results of gene expression levels in different samples of *R. ferrugineus*.

Supplementary material Figure S2：The volcanic map was used to represent the overall distribution of different genes with different insect states.

Figure. S2A. Volcano map of differentially expressed genes in Pupa and larva.

Figure. S2B. Volcanic map of differentially expressed genes in Pupa and male adults.

Figure. S2C. Volcanic map of differentially expressed genes in Pupa and female adults.

Figure. S2D. Volcanic map of differentially expressed genes in male adults and female adults.

Note: The scatter in the map represented each gene, the blue dot represented the gene with no significant difference, the red dot represented the up-regulated gene with significant difference, and the green dot represented the down-regulated gene with significant difference.

Supplementary material Figure S3-S5：H-cluster, k-means and SOM were used to cluster the relative expression level of differentially expressed genes log2(ratios).

Figure. S3. H-cluster was used for cluster cluster analysis.

Figure. S4. k-means was used for cluster cluster analysis.

Figure. S5. SOM was used for cluster cluster analysis.

Note: The gray line in each figure represents a broke-line graph of relative expression levels of genes in a cluster at different development stages, and the blue line represents a broke-line graph of the average relative expression levels of all genes in this cluster. The red line is for reference, the online is for up-regulation, and the offline is for down-regulation. The X-axis represents different developmental stages, and the Y-axis represents the relative expression of genes.

Supplementary material Figure. S6： The number of Long noncoding RNAs (LncRNAs) changes by comparing four different combinations. (Blue, downregulated Red, upregulated)

Figure. S6A. Volcano plot of DEGs predicted LncRNAs in combinations of Pupa and larva.

Figure. S6B. Volcano plot of DEGs predicted LncRNAs in combinations of Pupa and female adults.

Figure. S6C. Volcano plot of DEGs predicted LncRNAs in combinations of Pupa and male adults.

Figure. S6D. Volcano plot of DEGs predicted LncRNAs in combinations of male adults and female adults.
