# Supplementary figures and images for: Transcriptome and gene expression analysis of Rhynchophorus ferrugineus (Coleoptera: Curculionidae) during developmental stages
Source: PeerJ. 2020 Nov 2;8:e10223. doi: 10.7717/peerj.10223 (PMC7643551; doi:10.7717/peerj.10223)

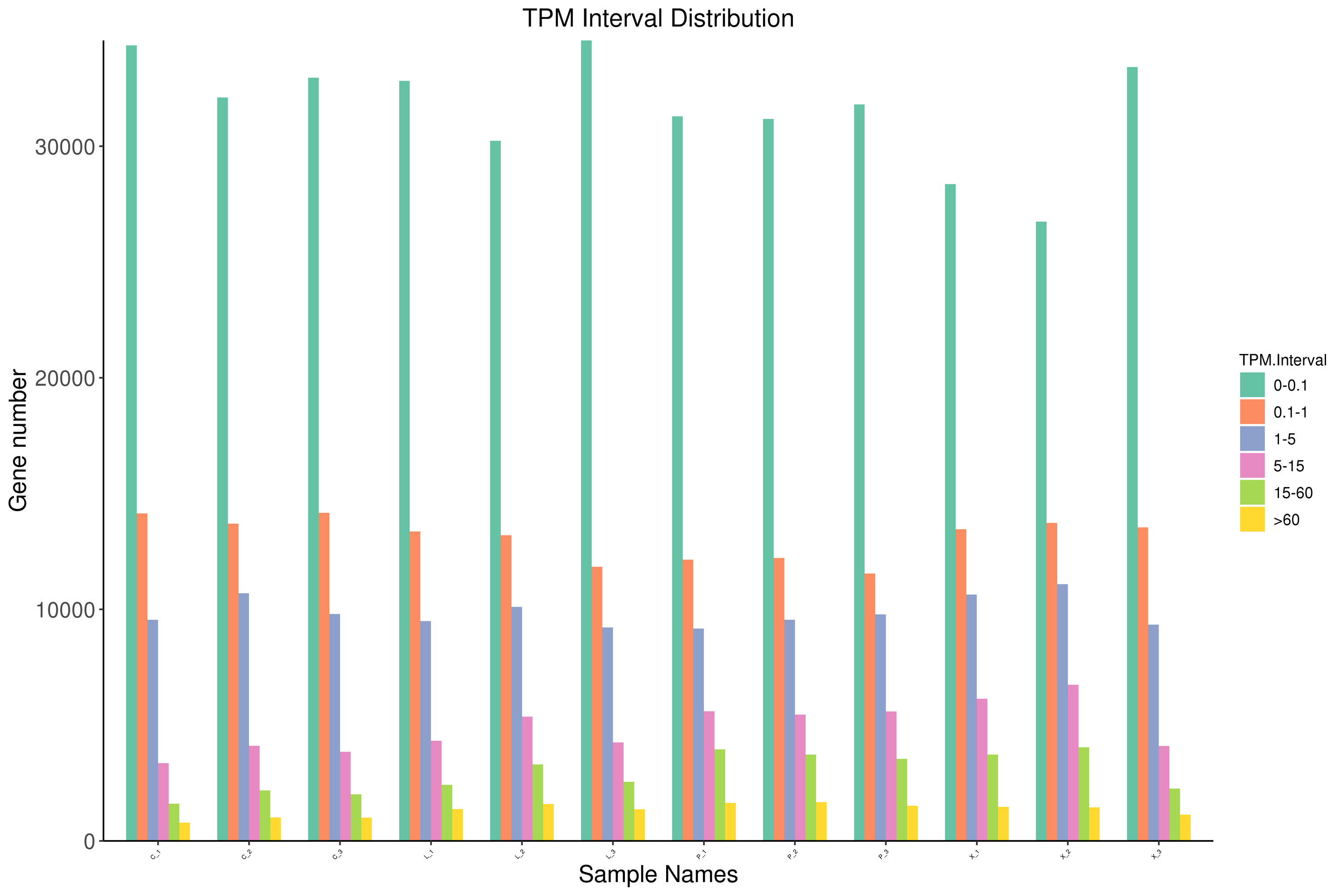

Supplement: Supplemental Information 1 [file peerj-08-10223-s001.zip › Supplemental Files/Supplementary Figures/Supplementary Figure S1.png]

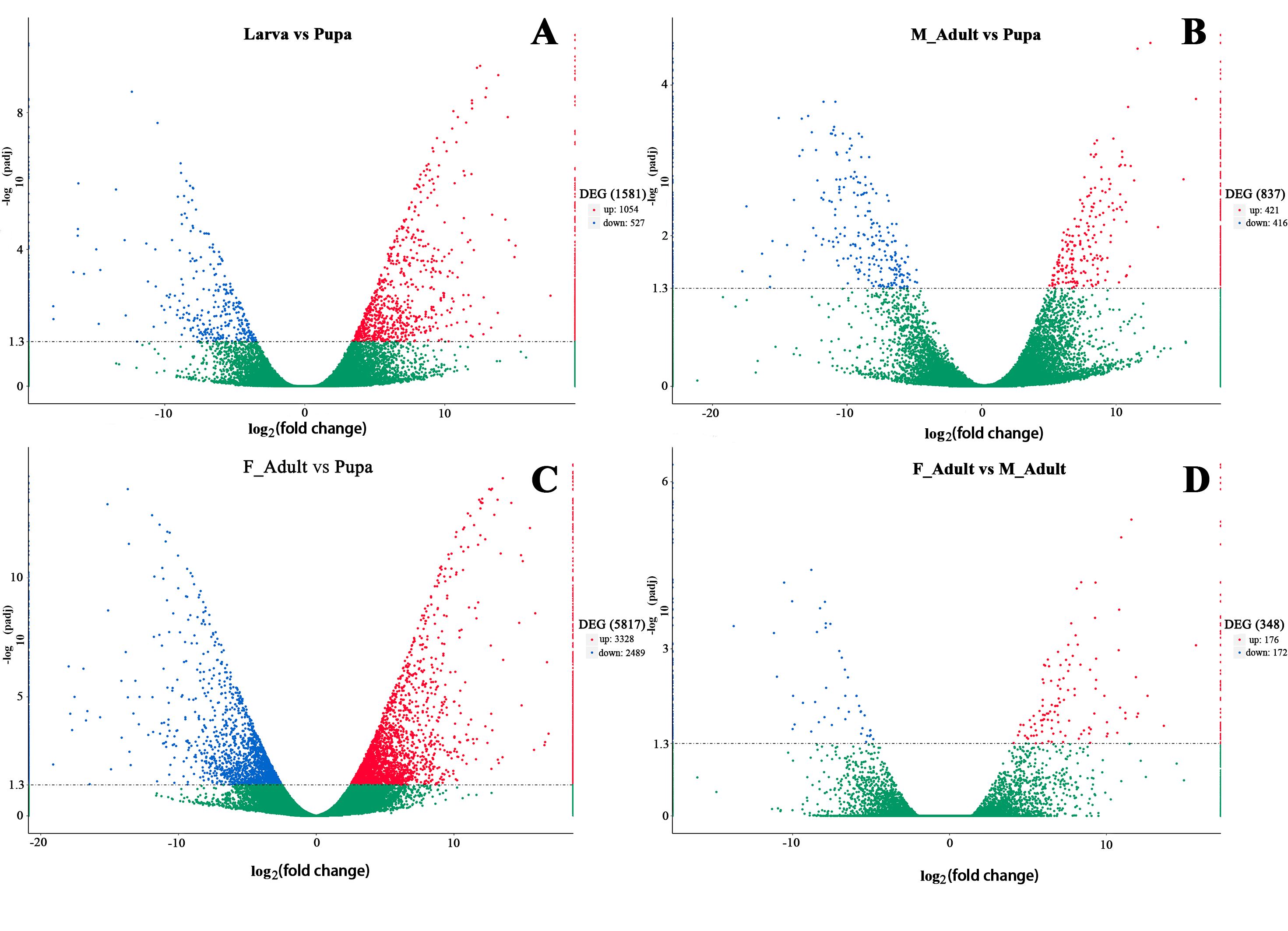

Supplement: Supplemental Information 1 [file peerj-08-10223-s001.zip › Supplemental Files/Supplementary Figures/Supplementary Figure. S2.png]

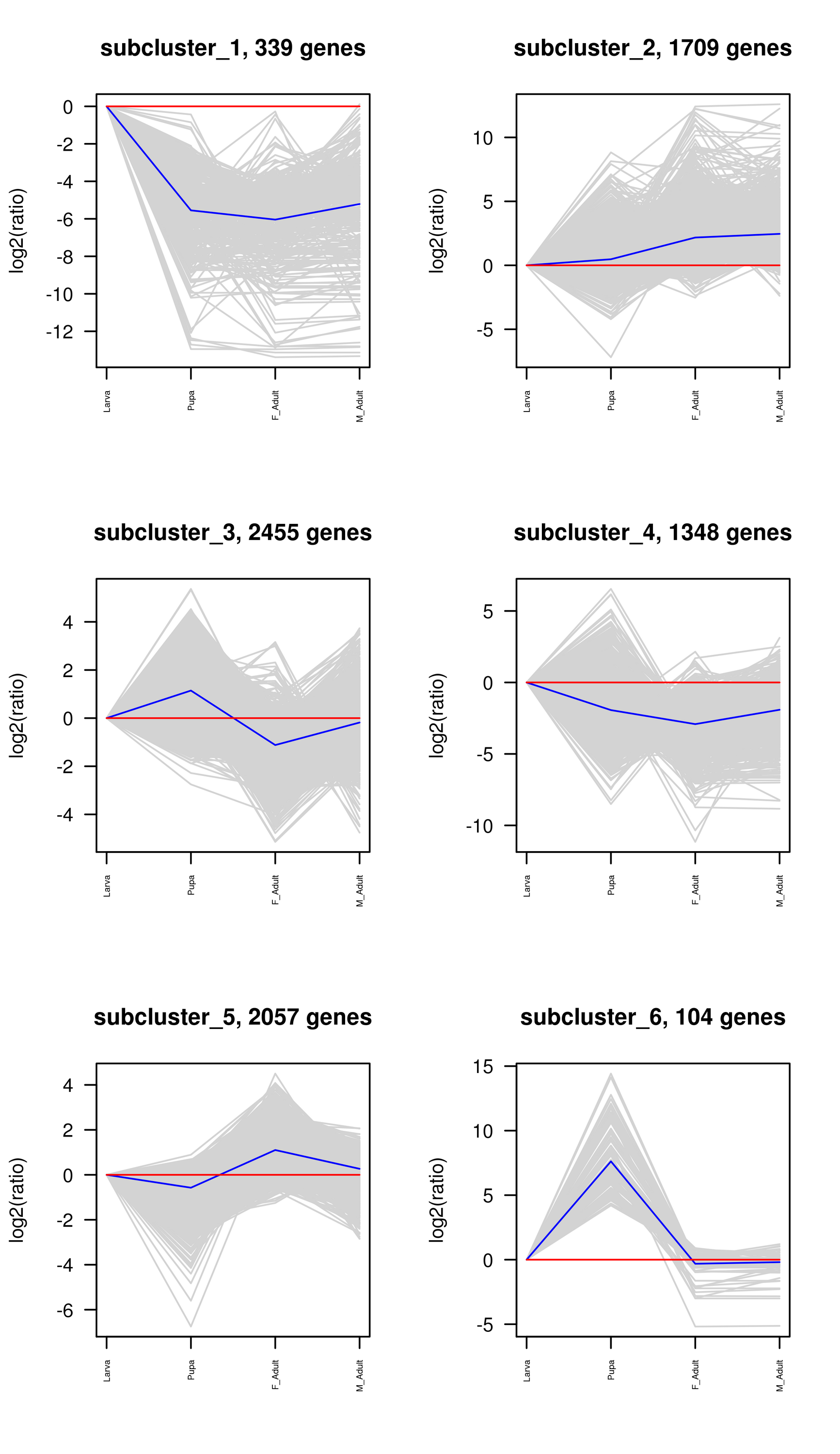

Supplement: Supplemental Information 1 [file peerj-08-10223-s001.zip › Supplemental Files/Supplementary Figures/Supplementary Figure. S3.png]

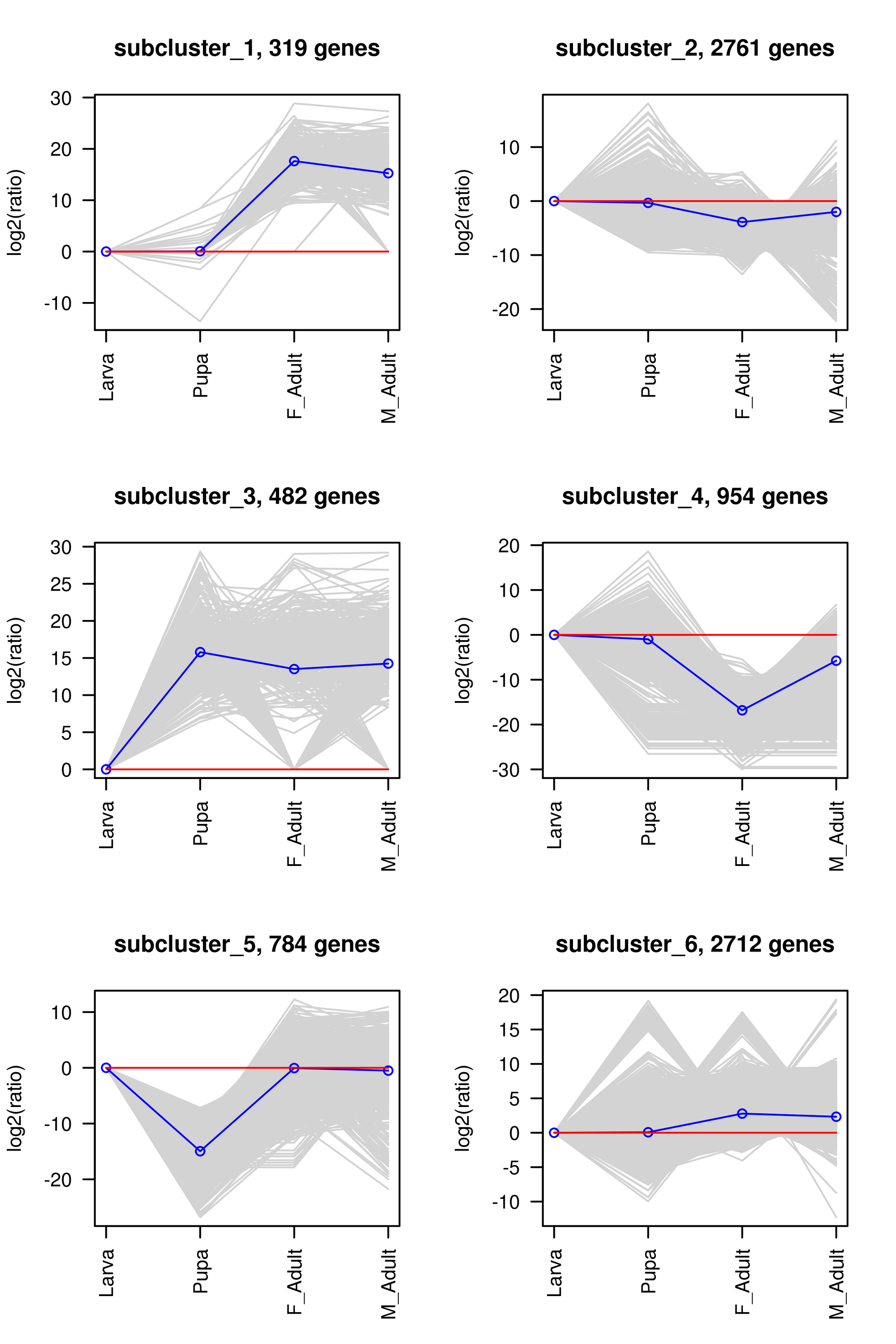

Supplement: Supplemental Information 1 [file peerj-08-10223-s001.zip › Supplemental Files/Supplementary Figures/Supplementary Figure. S4.png]

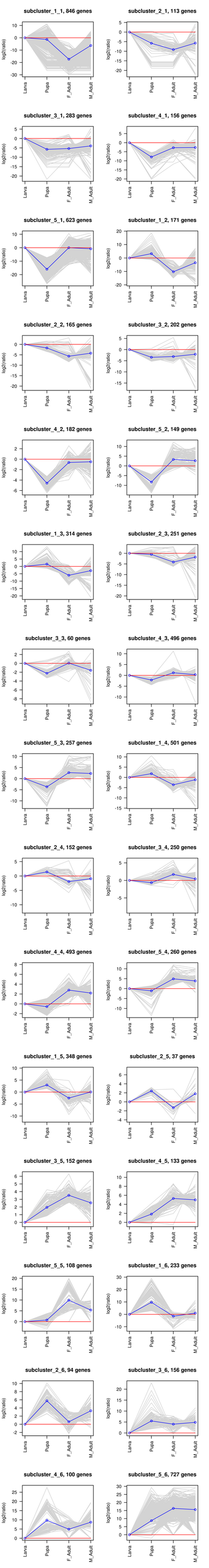

Supplement: Supplemental Information 1 [file peerj-08-10223-s001.zip › Supplemental Files/Supplementary Figures/Supplementary Figure. S5.png]

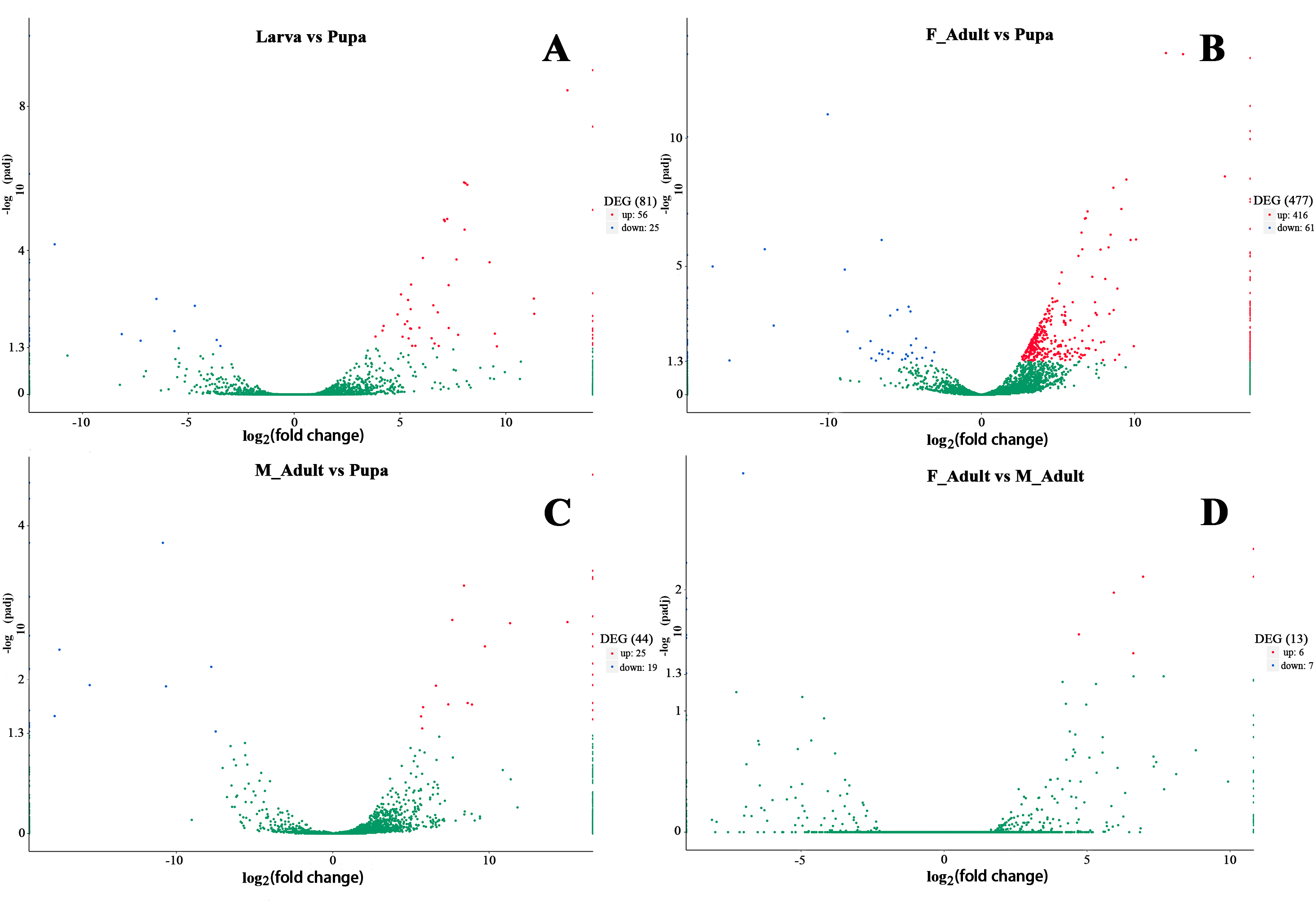

Supplement: Supplemental Information 1 [file peerj-08-10223-s001.zip › Supplemental Files/Supplementary Figures/Supplementary Figure. S6.png]
